# Cricoarytenoiditis as an Initial Manifestation of Systemic Lupus Erythematosus

**DOI:** 10.1155/2011/317379

**Published:** 2011-09-07

**Authors:** Jean-Michel Hougardy, Nicolas Roper, Alain Michils, Muhammad S. Soyfoo

**Affiliations:** ^1^Department of Internal Medicine, Erasme Hospital, Université Libre de Bruxelles, 1070 Brussels, Belgium; ^2^Department of Otho-Rhino-Laryngology, Erasme Hospital, Université Libre de Bruxelles, 1070 Brussels, Belgium; ^3^Department of Pneumology, Erasme Hospital, Université Libre de Bruxelles, 1070 Brussels, Belgium; ^4^Department of Rheumatology, Erasme Hospital, Université Libre de Bruxelles, 1070 Brussels, Belgium

## Abstract

Systemic lupus erythematosus (SLE) is a chronic inflammatory disease encompassing a broadened spectrum of clinical manifestations. Vocal cords involvement in SLE is not a frequent entity but can be life threatening if not treated. We hereby report the case of a patient presenting with cricoarytenoiditis and vocal cord dysfunction revealing SLE.

## 1. Introduction

Systemic lupus erythematosus (SLE) is a chronic inflammatory autoimmune disorder, which is characterized by systemic organ damage [1]. As such, SLE has no typical clinical presentation but most common features include fatigue, malaise, low-grade fever, anorexia, and lymphadenopathy. The musculoskeletal system is one of the most common organ system involved with arthralgia, tendinitis, myositis, or avascular necrosis [2]. Involvement of arytenoid joints during the course of SLE is a rare entity but can be potentially life threatening, entailing vocal cord dysfunction and ensuing respiratory distress. We hereby report the case of a patient presenting vocal cord dysfunction as an initial presentation of SLE.

## 2. Case Report

**Figure 1:**
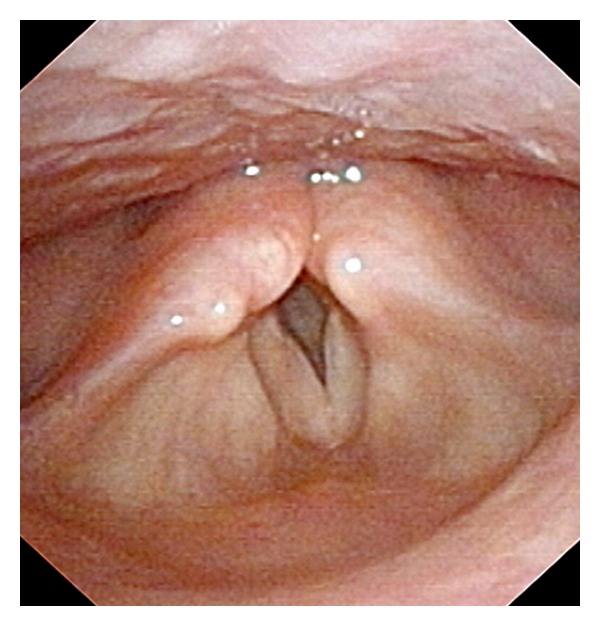
Nasofibroscopic view of the larynx in maximum abduction showing left vocal cord immobility in adduction.

A 20-year-old Caucasian man with no prior medical history was admitted to the emergency department for hoarseness, stridor, and pyrexia. The patient did not take any specific medications. Symptoms developed over a period of 2-3 months before admission and were associated with exhaustion, gonalgia, and depression. Three months before the current situation, the patient had undergone severe psychological stress. Physical examination revealed numerous submandibular polyadenopathy, attenuated heart sounds, and bilateral pulmonary crackles. Chest X-ray revealed cardiomegaly, and cardiac ultrasonography confirmed the presence of a 400 ml circumferential pericardial effusion and a thickened mitral valve but no hemodynamic changes. Initial laboratory findings showed increased C-reactive protein level at 4.5 mg/dl, normocytic anemia with hemoglobin of 9.2 g/dl, low serum albumin at 1.9 mg/dl, serum urea at 55 mg/dl, and serum creatinine at 1.0 mg/dl. The 24-hour urine collection showed increased proteinuria of 11 g. Autoimmune evaluation tests revealed increased antinuclear autoantibodies at a titre of 1 : 5000 and the presence of high titres of anti-dsDNA as well as the anti-Sm autoantibodies. The complement levels (C3 and C4) were diminished, and the Coombs test was positive. Anti-EBV and anti-CMV IgG were identified in absence of specific IgM. The patient was HIV seronegative. A renal biopsy was performed, and histological analysis revealed focal extramembranous proliferative glomerulonephritis. Cervical computed tomography and laryngoscopy showed left vocal cord paresis as well as hypomobility of the right vocal cord (Figure 1). The major involvement of both vocal cords required urgent endotracheal intubation followed by tracheotomy. Chest CT-scan showed bilateral pleural effusions and confluent opacities within the left pulmonary lobe. Recurrent nerve compression was reasonably excluded in the absence of mediastinal adenopathies. Fine-needle aspiration (FNA) was performed in a left submandibular adenopathy and revealed cytological features suggestive of follicular and paracortical lymph node hyperplasia without any signs of malignancy or granuloma. Cytologic examination of one of the samples obtained from bronchoalveolar lavage revealed numerous acid-fast bacilli. Because of high suspicion of active tuberculosis (TB), anti-TB chemotherapy (Isoniazid, rifampicin, pyrazinamide, and ethambutol) was started in association with glucocorticosteroids (pulse corticoids 1 g/day for 3 days). The patient's condition improved within a few days with the disappearance of vocal cord palsy. However, additional investigations (auramine staining and cultures of BAL and pleural fluids) as well as sustained cultures of the FNA of the adenopathy sample remained negative for mycobacterium, making the diagnosis of TB unlikely. The above clinical vignette encompassing serositis, glomerulonephritis, increased titres of antinuclear antibodies, the presence of antidsDNA, anti-Sm autoantibodies, and autoimmune anemia is typical of SLE. In the light of this atypical presentation, the vocal cord dysfunction was attributed to laryngeal involvement due to SLE.

As recurrent nerve palsy was reasonably ruled out, the diagnosis of cricoarytenoiditis was retained to explain the vocal cord dysfunction. Accordingly, immunosuppressive therapy including glucocorticoids (methylprednisolone 64 mg/d) and mycophenolate mofetil (2 g/d) was started.

## 3. Discussion

We report the case of SLE with an unusual presentation including respiratory distress due to laryngeal involvement. In addition, the presence of polyserositis associated with a positive auramine staining on submandibular FNA adenopathy samples were temporarily misleading. SLE and TB are not rare in Europe, and the clinical presentation of both diseases could be misleading. Active TB mimics lots of diseases, including SLE. Pericardial and pleural effusions associated with cervical polyadenopathy are well known to be classical findings of active TB in young adults [3]. Moreover, up to 30% of active TB are associated with significant titres of circulating ANA even those ANA are often uncharacterized [4, 5]. In this case, the presence of AFB within the initial cervical adenopathy FNA was additionally confusing. However, the absence of usual cytological features of TB (i.e., granuloma, caseous necrosis, or Langhan's giant cells) and the finally negativity of cultures of additional samples (BAL, pleural fluids, and biopsy of axillary adenopathy) led us to conclude for sample contamination as it was reported and to reconsider the diagnosis of active TB [6].

The rapid clinical improvement observed in our patient after initiation of anti-TB chemotherapy could be reasonably attributed to the adjunction of glucocorticoids.

Nearly all organs can be affected during SLE, the musculoskeletal system being the most often involved [1, 7]. Accordingly, inflammation of cricoarytenoid joints could be considered as a particular form of musculoskeletal involvement during SLE. Moreover, as laryngeal involvement occurs during SLE, the glottis and crycoarytenoid joints are the most often involved sites [8]. In the few published series, laryngeal involvement occurs in up to 30% of SLE patients [8, 9]. Laryngeal electromyography (EMG) can be useful in separating mechanical from neurogenic causes of vocal fold immobility. Conditions such as cricoarytenoid arthritis or arytenoids dislocation generally yield near normal EMG. Therefore, when laryngeal EMG is available, it should be considered, as it will become an integral part of the investigation of neurologic disorders affecting the larynx. Reported laryngeal lesions are numerous including arthritis of the cricoarytenoid joint, vocal fold hyperplasia, mucosal nodularity, ulceration, inflammation, edema, as well as necrotizing vasculitis with airway obstruction [8, 9]. Clinical manifestations are not specific with hoarseness, throat pain, dysphagia, cough, dyspnea, and even stridor. Thus, isolated laryngeal involvement could be misleading for the establishment of SLE diagnosis. Lastly, cranial neuropathy in the setting of neurological systemic lupus might as well be responsible for laryngeal nerve palsy and should also be searched for in the context of the aforementioned case. In our case, cranial neuropathy was reasonably excluded based on clinical examination and absence of clinical evidence of neurolupus.

In conclusion, this case underlines the need to exclude underlying SLE in the setting of unexplained laryngeal involvement. Accordingly, early detection and treatment of SLE could be associated with most favourable outcome [10].
